# Visit-to-visit variability of glycemia and vascular complications: the Hoorn Diabetes Care System cohort

**DOI:** 10.1186/s12933-019-0975-1

**Published:** 2019-12-12

**Authors:** Roderick C. Slieker, Amber A. W. H. van der Heijden, Giel Nijpels, Petra J. M. Elders, Leen M. ’t Hart, Joline W. J. Beulens

**Affiliations:** 1 Department of Epidemiology and Biostatistics, Amsterdam Public Health Institute, Amsterdam UMC, Location VUMC, De Boelelaan 1089a, 1081 HV Amsterdam, The Netherlands; 2 Department of Cell and Chemical Biology, Leiden University Medical Center, Leiden, The Netherlands; 3 Department of General Practice and Elderly Care Medicine, Amsterdam Public Health Institute, Amsterdam UMC, Location VUmc, Amsterdam, The Netherlands; 4 Molecular Epidemiology Section, Department of Biomedical Data Sciences, Leiden University Medical Center, Leiden, The Netherlands; 5 Julius Center for Health Sciences and Primary Care, University Medical Center Utrecht, Utrecht, The Netherlands

**Keywords:** Complications, Glycemia, Type 2 diabetes, Variability

## Abstract

**Background:**

Glycemic variation has been suggested to be a risk factor for diabetes-related complications. Previous studies did not address confounding of diabetes duration, number of visits and length of follow-up. Here, we characterize glycemic variability over time and whether its relation to diabetes-related complications and mortality is independent from diabetes- and follow-up duration.

**Materials and methods:**

Individuals with type 2 diabetes (n = 6770) from the Hoorn Diabetes Care System cohort were included in this study. The coefficient of variation (CV) was calculated over 5-year sliding intervals. People divided in quintiles based on their CV. Cox proportional hazard models were used to investigate the role of glycemic CV as risk factor in diabetes-related complications and mortality.

**Results:**

The coefficient of variation of glucose (FG-CV) increased with time, in contrast to HbA1c (HbA1c-CV). People with a high FG-CV were those with an early age of diabetes onset (Δ_Q5–Q1_ = − 2.39 years), a higher BMI (Δ_Q5–Q1_ = + 0.92 kg/m^2^), an unfavorable lipid profile, i.e. lower levels of HDL-C (Δ_Q5–Q1_ = − 0.06 mmol/mol) and higher triglycerides (Δ_Q5–Q1_ =+ 1.20 mmol/mol). People with the highest FG-CV in the first 5-year interval showed an increased risk of insulin initiation, retinopathy, macrovascular complications and mortality independent of mean glycemia, classical risk factors and medication use. For HbA1c, the associations were weaker and less consistent.

**Conclusions:**

Individuals with a higher FG-CV have an unfavorable metabolic profile and have an increased risk of developing micro- and macrovascular complications and mortality. The association of HbA1c-CV with metabolic outcomes and complications was less consistent in comparison to FG-CV.

## Background

People with type 2 diabetes have a 2–4 times increased risk to develop macrovascular complications [1, 2]. Therefore, an important challenge for diabetes-related micro- and macrovascular complications is to identify new risk factors.

Over the past years, an increasing number of studies have investigated visit-to-visit glycemic variability as risk factor for diabetes-related complications and its underlying mechanism [3–17]. These studies used a measure of variation of fasting glucose or HbA1c over the follow-up duration to assess glycemic variability and showed that a high glycemic variability is a risk factor for micro- and macrovascular complications and mortality independent of their respective means [3–15]. This increased risk could be explained by associated cardiometabolic risk factors and even cardiac structure [18, 19], but studies characterizing the relation between glycemic variability and anthropomorphic, metabolic and lifestyle factors are sparse. Only Noyes et al. investigated this relation and showed a high glycemic variability, defined as the CV and standard deviation, occurred more often in more intensively treated individuals with a low mean HbA1c [20]. Moreover, a high HbA1c variability was associated with male sex, a younger age, a lower HDL-C and a higher BMI [20].

Thus far, studies investigating variability in relation to complications [3–13] or individuals’ characteristics have mostly used the same approach of calculating variability over the total follow-up time in which people differed in their time since diagnosis, follow-up time, number of visits and the distribution of visits across the follow-up [20]. This approach has several limitations. Individuals with the highest variability are more likely to have a longer time since diagnosis [21, 22]. Also, people with more visits, a higher frequency of visits, and a long follow-up are more likely to have a high variability. More visits and higher frequency in a short period of time are most likely driven by poor glycemic control leading to an increased glycemic variability. This will therefore confound the association and as such the interpretation between glycemic variability and diabetes-related complications. Finally, by combining all follow-up measurements, information is lost about the changes in variability over time.

By investigating the glycemic variability across fixed time intervals of follow-up, aligned with time since diagnosis, with equal time between measurements, one could overcome these confounding factors. We here investigate the relation of glycemic variability over time with clinical characteristics, medication usage and micro- and macrovascular complications and mortality in the Hoorn Diabetes Care System (DCS) cohort, a large prospective cohort of people with type 2 diabetes with annual repeated measures of risk factors and complications.

## Methods

### Study population

People from the Hoorn DCS cohort were included, which is an open prospective cohort of people with type 2 diabetes from the northwest part of the Netherlands [23]. People were diagnosed with type 2 diabetes when they had fasting glucose level ≥ 7 mmol/l on two separate days, or fasting glucose level ≥ 7 mmol/l with hyperglycemic symptoms or any glucose level ≥ 11 mmol/l [24]. As part of routine care, individuals visit the DCS once a year for diabetes monitoring [23].

### Selection

Data of 12,869 individuals (Data freeze 1998–2015) were used for analysis (Additional file 1: Figure S1A). Individuals were excluded when they only had visits within 6 months after diagnosis (N = 1082), a follow-up shorter than 5 years (N = 4775), an age of diabetes onset below 35 years to exclude those with type 1 diabetes (N = 229) or those with missing values (N = 3). In total, 6780 individuals were included for analysis (Additional file 1: Figure S1A).

### Clinical characteristics

Smoking status was self-reported. Systolic and diastolic blood pressure were measured twice at each visit 3 min apart with the average used for analysis [23]. Current medication use was registered based on the dispensing labels of the medication. All laboratory measurements were measured in a fasted state. All HbA1c measurements were performed using the turbidimetric inhibition immunoassay for hemolyzed whole EDTA blood (Cobas c501, Roche Diagnostics, Mannheim, Germany, run CV 1.6%) [23], fasting glucose levels were measured in fluorinated plasma using hexokinase with the UV test (Cobas c501, Roche Diagnostics, run CV 1.3%) [23]. Levels of triglycerides (mmol/L), total cholesterol (mmol/L), HDL-C (mmol/L) and serum creatinine levels were measured enzymatically (Cobas c501, Roche Diagnostics). LDL-C levels (mmol/L) were derived according from cholesterol- and triglycerides levels [25].

### Glycemic variability

Visits between diagnosis and the first 6 months after diagnosis date were excluded, to reduce the effect on variability of the initial treatment. Glycemic variability was calculated over 5-year intervals, with five measurements 1 year apart (Additional file 1: Figure S1b). Variability in FG and HbA1c for the first 20 intervals was calculated as the standard deviation, median absolute deviation and the coefficient of variation (CV). Five-year intervals were aligned based on time since diagnosis, i.e. the first interval represents 1–5 years after diagnosis. Not all individuals were in all intervals, where individuals were allowed to be in a subset of the intervals. Only intervals 1–20 with ≥ 200 individuals were considered (Interval 1–20, Additional file 2: Table S1). Individuals within an interval were split into quintiles based on their CV (1 = low CV, 5 = high CV).

### Progression outcomes

Insulin initiation was defined as treatment with insulin for two subsequent years or the requirement to initiate insulin treatment but without actual treatment. Insulin requirement was defined as HbA1c levels over > 8.5% or 69 mmol/mol for two subsequent years while treated with two or more oral glucose-lowering drugs.

Retinopathy state was based on annual fundus photography of both eyes and graded according to the EURODIAB classification score. Time to referable retinopathy was defined as the time between diagnosis and the first occurrence of stage 2: moderate non-proliferative retinopathy or higher [23].

Annual estimated glomerular filtration rate (eGFR) was calculated using the CKD-EPI formula for people of European descent [26]. Based on CKD-EPI eGFR, eGFR stages were defined as: (1) normal or high (≥ 90 mL/min/1.73 m^2^), (2) mildly decreased (60–89), (3a) mildly to moderately decreased (45–59), (3b) moderately to severely decreased (30–44), (4) severely decreased (15–29) and (5. kidney failure (< 15) [27]. The first date at which stage 4 or an eGFR < 29 mL/min/1.73 m^2^ was reached was used as the eGFR endpoint [27].

Stages of chronic kidney disease (CKD) were defined as previously described [27, 28]. Endpoint was the first date at CKD stage 2 or 3, which are people with the following combination:

- eGFR stage 1/2 and albuminuria > 30 mg/mmol
- eGFR stage 3a and albuminuria 3–30 mg/mmol
- eGFR stage 3b and albuminuria < 3 mg/mmol
- eGFR stage 4/stage 5

Urinary albumin was measured turbidimetrically (Cobas c501, Roche Diagnostics) and creatinine levels enzymatically (Cobas c501, Roche Diagnostics) as described elsewhere [23]. Micro- and microalbuminuria were defined as the first visit at which the urinary albumin/creatinine ratio was over 2.5 mg/mmol (male)/3.5 mg/mmol (female) and 25 mg/mmol (male)/35 mg/mmol (female), respectively.

Onset of macrovascular complications was defined as the time upon the first macrovascular event relative to diagnosis. Macrovascular complications were self-reported, but all self-reported events were verified against electronic medical registrations from the regional hospital and GP [23]. We validated this procedure in a subsample of 453 participants showing a sensitivity of 86% and a specificity of 90% [23]. ICD-9 410–449 or ICD-10 I10–I79 were included for macrovascular complications, including ischemic heart disease, diseases of pulmonary circulation, other forms of heart disease, cerebrovascular disease, cerebrovascular disease and diseases of arteries, arterioles, and capillaries. Vital status was checked every 6 months using the National population registry [23].

### Statistical analysis

Associations between glycemic variability and continuous determinants were performed using a linear regression with adjustment for sex, age at diagnosis and mean glucose or HbA1c. Associations between glycemic variability and dichotomous determinants were performed using logistic regression adjusted for the same covariates. A model was run for each of the 20 intervals on each of the determinants. *P*-values were adjusted for multiple testing using the Bonferroni procedure, with *P*-values ≤ 2.5·10^−3^ (0.05/20 intervals) considered significant.

Cox proportional hazard models were performed to test glycemic variability as risk factor for diabetes progression. Time to event was defined as the time between diagnosis date and date of first event of micro- and macrovascular complications or death. Only first interval was investigated in Cox PH models to avoid survival bias and models were stratified by glucose or HbA1c tertiles. The crude model was adjusted for age at diagnosis, mean BMI and sex. The fully adjusted model additionally included mean HDL and triglycerides, first HbA1c, oral glucose lowering drugs use, insulin use and eGFR (eGFR, CKD, micro- and macroalbuminuria models). The lowest quintile of glycemic variability was used as the reference group. A trend test across glycemic variability quintiles was performed by including the median coefficient of variation of fasting glucose (FG-CV) and HbA1c (HbA1c-CV) of each quintile as continuous variable in the model. Individuals with missing data in the models were removed (< 0.5%). All analyses were performed with R statistics (version 3.5.1) in combination with the R packages *survival* (2.43–3) and *ggplot2* (version 3.1.0).

## Results

Clinical characteristics of the participants in the first interval are shown in Table 1. Time since diagnosis was on average 1.1 years (IQR = 0.9–1.2 years, Table 1). Both the coefficient of variation of fasting glucose (FG-CV) and HbA1c (HbA1c-CV) showed the lowest correlation with the mean fasting glucose and HbA1c, respectively (r_FG_ = 0.42 and r_HbA1c_ = 0.49), in contrast to the standard deviation and the median absolute deviation and where therefore used to reduce the effect of mean glycemia.

**Table 1:** Baseline characteristics of the 3963 individuals within the first interval for FG-CV quintiles

Outcome	All with first interval			Q1			Q2			Q3			Q4			Q5			P value
	Mean	CI25	CI75	Mean	CI25	CI75	Mean	CI25	CI75	Mean	CI25	CI75	Mean	CI25	CI75	Mean	CI25	CI75	
N	3963			792			793			792			793			793			
Sex (%)	53			51			52			50			54			56			0.10
Age (years)	61.6	54.2	69.3	63.5	56.6	70.4	63.4	56.6	70.9	61.6	55	68.9	60.2	52.6	68.2	59.4	50.9	67.7	≤ 0.001
Time since diagnosis	1.1	0.9	1.2	1.1	0.9	1.2	1.1	1	1.2	1.1	0.9	1.2	1.1	0.9	1.2	1.1	0.9	1.2	≤ 0.001
BMI (kg/m^2^)	30.1	26.5	32.9	29.1	25.9	31.6	30	26.5	32.8	30.2	26.5	32.9	30.4	26.7	33.5	30.8	26.7	33.8	≤ 0.001
HbA1c (%)	6.7	6	7	6.3	5.9	6.6	6.4	6	6.7	6.6	6.1	6.9	6.8	6.1	7.2	7.6	6.4	8.3	≤ 0.001
HbA1c (mmol/mol)	50.0	42.5	53	45.2	41	48.6	46.3	42	49.7	48.3	43	51.9	50.8	43.2	55	59.3	46	67.2	≤ 0.001
Glucose (mmol/L)	7.8	6.7	8.4	7.1	6.4	7.6	7.4	6.6	8.1	7.6	6.7	8.2	7.8	6.8	8.7	8.9	7	10.2	≤ 0.001
HDL (mmol/L)	1.2	1	1.4	1.3	1.1	1.5	1.3	1	1.5	1.2	1	1.4	1.2	1	1.4	1.2	1	1.3	≤ 0.001
LDL (mmol/L)	3.0	2.2	3.6	2.9	2.2	3.6	3	2.2	3.6	3	2.2	3.6	3	2.2	3.6	3	2.3	3.6	0.39
Triaglycerol (mmol/L)	1.9	1.2	2.2	1.7	1.1	2	1.8	1.2	2.1	1.9	1.2	2.2	1.9	1.2	2.3	2.2	1.3	2.5	≤ 0.001
SBP (mmHg)	141.7	128	154	141.7	128	153	143.5	130	156	142.2	128	154	140.8	126.2	153	140.2	127	152	0.01
DBP (mmHg)	80.1	73	86	79.7	73	85	79.7	73	86	79.9	73	87	80.7	74	87	80.7	74	88	≤ 0.01
Metformin (%)	54			48			53			52			58			58			0.01
Sulfonylureas (%)	25			14			18			20			31			42			≤ 0.001
Insulin (%)	5			2			2			3			5			12			≤ 0.001
Other glucose lowering drugs (%)	2			1			2			1			2			2			0.26
ACE	50.1			50.4			52.8			49.1			51.3			50.4			0.65
ARB	73.9			75.0			74.1			75.4			74.7			70.2			0.13
Smoking, % yes	19.9			16.2			16.0			19.7			21.6			26.0			≤ 0.001
% no	67.1			68.9			68.6			67.3			66.3			64.1			
% former	10.8			12.1			13.0			10.6			10.6			7.8			
% unknown	2.2			2.8			2.4			2.4			1.5			2.1			
European descent (%)	90.4			92.9			91.4			89.7			90.3			87.9			0.09

At baseline, individuals with a high FG-CV had on average a significantly higher BMI (Q1 = 29.1 vs Q5 = 30.8 kg/m^2^), higher fasting glucose (Q1 = 7.1, Q5 = 8.9 mmol/L) and higher triglycerides (Q1 = 1.7, Q5 = 2.2 mmol/L, Table 1). The proportion current smokers was higher in the highest quintile group (Q5 = 26.0% vs Q1 = 16.2%). Individuals with a high FG-CV were more often on metformin (Q1 = 48%, Q5 = 58%), sulphonylureas (SU, Q1 = 14%, Q5 = 42%) and insulin (Q1 = 2%, Q5 = 12%, Table 1). The characteristics for HbA1c-CV quintiles resembled those of FG-CV (Additional file 3: Table S2).

### FG-CV increases over time in contrast to HbA1c-CV

**Fig. 1:**
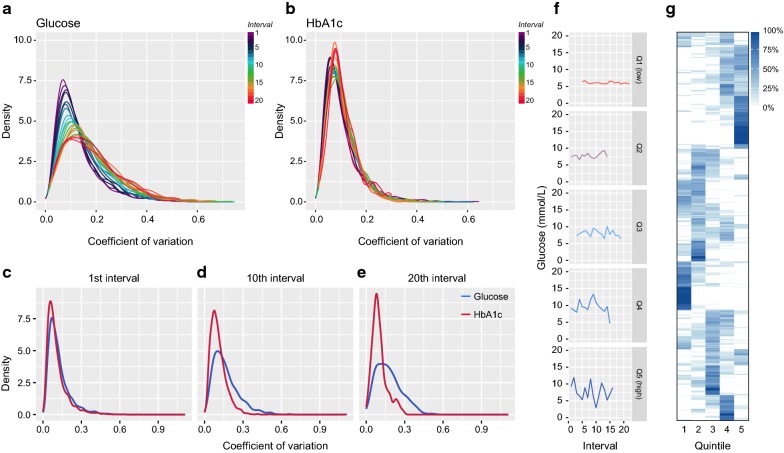
Glucose- and HbA1c-CV across time. Density plot of glucose-CV (**a**) and HbA1c (**b**) across the first 20 intervals. X-axis, level of FG-CV; y-axis: smoothed frequency; Colors represent the intervals with purple the 1st interval and red the 20th interval, density plot of the glucose-CV (blue) vs HbA1c-CV (red) in the first interval (**c**), tenth interval (**d**) and twentieth interval (**e**). **f** Example of five individuals that remained in the same quintile across all intervals across their follow-up. Each row represents one individual and each column the five quintiles. **g** Percentage of intervals compared to total number of individuals that ended up in a certain quintile, i.e. 100% means all intervals of an individual were assigned to that quintile

FG-CV increased in almost a linear fashion across the 5-year intervals (Fig. 1a, Additional file 4: Figure S2a), while HbA1c-CV remained largely stable over time (Fig. 1b–e). The correlation between HbA1c-CV and FG-CV also declined with time (Additional file 4: Figure S2b). In the first interval, individuals were often in the same interval for FG-CV and HbA1c-CV (Additional file 4: Figure S2d), although a substantial number was also in neighboring quintiles.

Between the lowest and the highest FG-CV quintiles, the differences in fasting glucose variability were clearly visible (Fig. 1f). Individuals with low or high FG-CV and HbA1c-CV largely remained in the same quintile across their follow-up (Fig. 1g and Additional file 4: Figure S2c). For FG-CV, 74.4% had at least 50% of their intervals in one quintile and 72.0% had at least 75% of their intervals in two adjacent quintiles.

### High FG-CV associates with higher triglycerides and BMI and lower HDL-C levels and an earlier disease onset

**Fig. 2:**
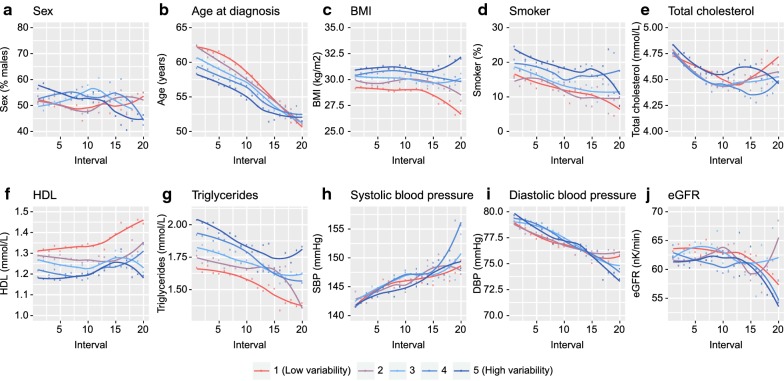
Characterization of individuals based on their FG-CV. Unadjusted outcome against the five quintiles with the fifth quintile being the group with the highest FG-CV against **a** sex (% males), **b** age at diagnosis, **c** BMI, **d** % smokers, **e** total cholesterol levels, **f** HDL-C levels, **g** triglycerides levels, **h** DBP, **i** SBP, **j** eGFR. Lines represent loess smoothed levels over 5-year average values of outcomes

The profile of people with a high FG-CV or HbA1C-CV was largely similar. For sex, no consistent difference was observed in FG-CV or HbA1c-CV (*P*_*bonf*_ > 0.05, Fig. 2a, Additional file 5: Table S3, Additional file 6: Table S4). Individuals with a high FG-CV and HbA1c-CV were those with an early diagnosis age (Q_2_ = 0.26, Q_5_ = − 2.39 years; Additional file 5: Table S3, Fig. 2b). A high FG-CV and HbA1c-CV was associated with a higher BMI in the first interval (Q_2_ = 0.76, Q_5_ = 0.92 kg/m^2^; Fig. 2b, Additional file 5: Table S3). The difference in BMI persisted over time for both FG-CV and HbA1c-CV. Finally, a high FG-CV was associated with lower HDL-C levels (Q_2_ = − 0.01, Q_5_ = − 0.06 mmol/L, Fig. 2b, Additional file 5: Table S3) and a higher level of triglycerides (Q_2_ = 1.02, Q_5_ = 1.07 mmol/L; Fig. 2g, Additional file 5: Table S3). The lower HDL-C levels and higher triglycerides in the highest FG-CV quintile persisted for most intervals (Fig. 2f, g) and resembled the results for HbA1c-CV (Additional file 7: Figure S3f, g, Table S4). For total cholesterol (Fig. 2e), blood pressure (Fig. 2h–i), eGFR (Fig. 2j), and smoking (Fig. 2d) no consistent associations were observed across FG-CV quintiles (Fig. 2e, h–j; Additional file 6: Table S4). A high HbA1c-CV was however, associated with a higher diastolic blood pressure (intervals 1–7, 9; *P*_bonf_ ≤ 0.04, but not SBP (Additional file 6; Table S4, Additional file 7: Figure S3). Of note, limiting the analysis to only the people that were included in the first interval did not change the result (data not shown).

### A high FG-CV is associated with more intense treatment and earlier insulin initiation

**Fig. 3:**
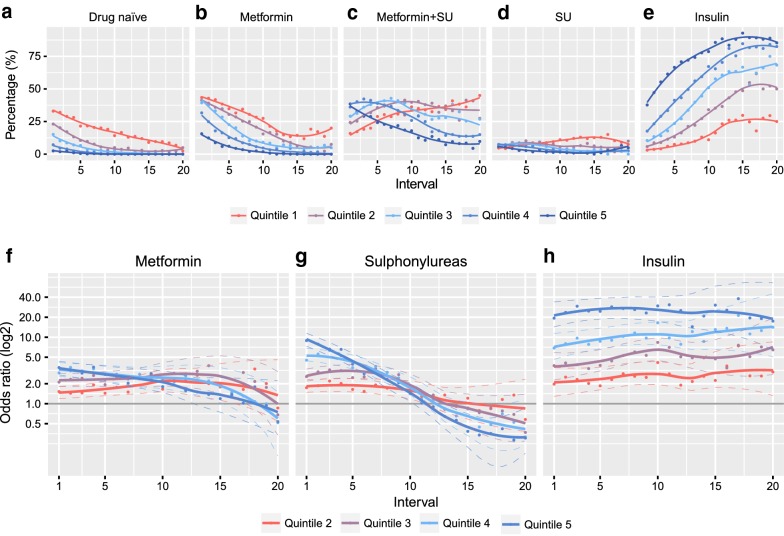
Glucose-lowering treatments across quintiles. Percentage of individuals per quintile untreated (**a**), on metformin only (**b**), combination of metformin and SU (**c**), SU monotherapy (**d**) and insulin (**e**). Odds ratios of four highest quintiles versus the lowest quintile across time for **f** metformin, **g** sulphonylureas and **h** insulin. *SU* sulphonylureas

Individuals with a high FG-CV and HbA1c-CV were different in how they were treated. A low proportion of individuals were drug-naïve in the highest FG-CV (2.5%) and HbA1c-CV (2.8%) versus the lowest quintile (33.0% and 34.6%, Fig. 3a). The proportion of individuals on dual therapy with both metformin and SU quickly decreased with time in the highest FG-CV and HbA1c-CV groups (38.5% and 42.4% to 9.8% and 19.5%), while in the lowest quintile the number of individuals treated with both metformin and SU increased (15.9% and 14.6% to 45% and 30%, Fig. 3b–d).

Relative to the lowest FG-CV quintile, those in the highest FG-CV quintile had a significantly higher use of metformin and SU use in the first 11 and nine intervals respectively (Fig. 3f, g, Additional file 8: Table S5). For HbA1c-CV similar observations were seen (Additional file 9: Figure S4).

Insulin use was much higher in the highest quintile versus the lowest quintile (Fig. 3e), across all 20 intervals with an OR of 19.37 (95% CI 12.86–30.55; *P*_*trend*_ = 4.11·10^−40^) in the first interval and 17.50 (95% CI 6.05–58.39; *P*_*trend*_ = 2.22·10^−7^) in the 20th interval (Fig. 3h, Additional file 8: Table S5). Individuals with a high FG-CV also went faster on insulin (Fig. 3e) with HRs ranging from 1.00 (Q_2_ 95% CI 0.75–1.33; *P* value = 0.98) to 3.33 (Q5 95% CI 2.61–4.27, *P *= 8.43·10^−22^) and *P*_trend_ = 3.31·10^−44^ (Table 2) in the fully adjusted model. For HbA1c-CV, lower hazards were observed than FG-CV, ranging from 1.52 (Q_2_ 95% CI 1.13–2.04, *P *= 5.38·10^−3^) to 2.45 (Q_5_ 95% CI 1.86–3.22, *P *= 2.00·10^−10^ and *P*_trend_ = 4.00·10^−11^).

**Table 2:** Hazard ratios of variability of fasting glucose and HbA1c for risk on insulin initiation, diabetes-related complications

	Fasting glucose CV						HbA1c–CV					
	Base			Adjusted			Base			Adjusted		
	HR (95% CI)	P-value	P_trend_	HR (95% CI)	P-value	P_trend_	HR (95% CI)	P-value	P_trend_	HR (95% CI)	P-value	P_trend_
Insulin initiation, N = 3955, events = 1116							N = 3955, events = 1116					
Q2	1.07 [0.80–1.43]	0.64	5.23·10^−67^	1.00 [0.75–1.33]	0.98	3.31·10^−44^	1.57 [1.17–2.10]	2.90·10^−3^	2.31·10^−17^	1.52 [1.13–2.04]	5.38·10^−3^	4.00·10^−11^
Q3	1.56 [1.20–2.03]	9.81·10^−4^		1.47 [1.13–1.91]	4.50·10^−3^		1.84 [1.39–2.44]	2.51·10^−5^		1.71 [1.29–2.28]	2.12·10^−4^	
Q4	2.43 [1.90–3.12]	2.54·10^−12^		2.10 [1.64–2.70]	5.70·10^−9^		2.53 [1.93–3.33]	2.37·10^−11^		2.39 [1.81–3.14]	5.31·10^−10^	
Q5	4.38 [3.44–5.59]	8.69·10^−33^		3.33 [2.61–4.27]	8.43·10^−22^		2.88 [2.19–3.78]	2.76·10^−14^		2.45 [1.86–3.22]	2.00·10^−10^	
Retinopathy N = 3893, events = 182							N = 3898, events = 182					
Q2	1.54 [0.75–3.17]	0.24	2.12·10^−9^	1.37 [0.67–2.82]	0.39	1.08·10^−3^	0.77 [0.39–1.52]	0.46	0.15	0.73 [0.37–1.44]	0.36	0.80
Q3	1.82 [0.91–3.65]	0.09		1.56 [0.78–3.14]	0.21		1.41 [0.78–2.55]	0.26		1.24 [0.68–2.25]	0.48	
Q4	3.08 [1.61–5.89]	6.85·10^−4^		2.35 [1.22–4.51]	0.01		1.53 [0.86–2.73]	0.14		1.28 [0.71–2.29]	0.41	
Q5	4.52 [2.39–8.57]	3.77·10^−6^		2.59 [1.34–5.01]	4.56·10^−3^		1.41 [0.79–2.52]	0.25		1.06 [0.59–1.92]	0.84	
eGFR stage N = 3959, events = 503							N = 3965, events = 507					
Q2	0.97 [0.73–1.29]	0.81	0.03	0.95 [0.71–1.27]	0.73	0.28	1.20 [0.90–1.61]	0.22	0.39	1.23 [0.92–1.66]	0.16	0.45
Q3	1.07 [0.79–1.44]	0.66		1.06 [0.78–1.43]	0.72		1.04 [0.77–1.41]	0.78		1.04 [0.77–1.40]	0.80	
Q4	1.03 [0.77–1.39]	0.82		0.91 [0.68–1.23]	0.55		1.09 [0.81–1.48]	0.56		1.16 [0.86–1.57]	0.34	
Q5	1.31 [0.98–1.76]	0.07		1.16 [0.85–1.58]	0.35		1.21 [0.89–1.66]	0.23		1.20 [0.87–1.65]	0.26	
CKD stage N = 2688, events = 802							N = 2694, events = 806					
Q2	0.92 [0.72–1.16]	0.46	5.52·10^−3^	0.92 [0.72–1.16]	0.46	0.05	1.08 [0.85–1.37]	0.51	0.61	1.10 [0.87–1.39]	0.42	0.73
Q3	0.92 [0.72–1.17]	0.50		0.92 [0.72–1.17]	0.50		0.90 [0.71–1.15]	0.41		0.88 [0.69–1.11]	0.28	
Q4	1.05 [0.83–1.32]	0.70		1.05 [0.83–1.32]	0.70		0.96 [0.75–1.22]	0.73		0.95 [0.74–1.22]	0.68	
Q5	1.23 [0.97–1.55]	0.08		1.23 [0.97–1.55]	0.08		1.07 [0.84–1.37]	0.58		1.05 [0.82–1.35]	0.69	
Microalbuminuria (N = 3949, events = 1448)							N = 3953, events = 1452					
Q2	1.25 [1.04–1.50]	0.01	4.23·10^−7^	1.20 [1.00–1.44]	0.05	0.01	1.04 [0.87–1.25]	0.65	1.43·10^−4^	1.01 [0.84–1.21]	0.94	0.01
Q3	1.39 [1.16–1.67]	3.33·10^−4^		1.32 [1.10–1.58]	2.54·10^−3^		1.15 [0.96–1.37]	0.13		1.09 [0.91–1.31]	0.33	
Q4	1.53 [1.28–1.83]	3.43·10^−6^		1.37 [1.15–1.65]	6.05·10^−4^		1.31 [1.09–1.56]	3.40·10^−3^		1.23 [1.03–1.47]	0.02	
Q5	1.63 [1.36–1.96]	1.33·10^−7^		1.36 [1.13–1.65]	1.51·10^−3^		1.38 [1.14–1.66]	7.11·10^−4^		1.23 [1.02–1.49]	0.03	
Macroalbuminuria N = 3949, events = 250							N = 3953, events = 251					
Q2	1.32 [0.80–2.17]	0.28	2.98·10^−7^	1.28 [0.77–2.12]	0.34	6.56·10^−4^	1.42 [0.89–2.25]	0.14	0.12	1.42 [0.89–2.27]	0.14	0.32
Q3	1.78 [1.09–2.89]	0.02		1.69 [1.03–2.75]	0.04		0.97 [0.59–1.59]	0.91		0.95 [0.58–1.57]	0.85	
Q4	1.97 [1.22–3.18]	0.01		1.79 [1.10–2.91]	0.02		1.35 [0.85–2.15]	0.21		1.29 [0.80–2.07]	0.29	
Q5	2.89 [1.82–4.60]	7.40·10^−6^		2.26 [1.39–3.70]	1.11·10^−3^		1.49 [0.93–2.40]	0.10		1.36 [0.84–2.21]	0.21	
Macrovascular complications N = 3437, events = 479							N = 3442, events = 480					
Q2	1.15 [0.83–1.58]	0.40	1.56·10^−3^	1.14 [0.83–1.57]	0.41	3.96·10^−3^	0.97 [0.70–1.34]	0.84	0.02	0.97 [0.70–1.34]	0.84	0.04
Q3	1.41 [1.03–1.93]	0.03		1.39 [1.01–1.91]	0.04		1.27 [0.94–1.73]	0.12		1.27 [0.94–1.73]	0.12	
Q4	1.43 [1.04–1.95]	0.03		1.41 [1.02–1.94]	0.04		1.27 [0.93–1.73]	0.13		1.27 [0.93–1.73]	0.13	
Q5	1.64 [1.20–2.24]	2.10·10^−3^		1.62 [1.16–2.26]	4.28·10^−3^		1.39 [1.01–1.91]	0.05		1.39 [1.01–1.91]	0.05	
All–cause mortality N = 3959, events = 691							N = 3965, events = 696					
Q2	1.00 [0.77–1.31]	0.99	7.31·10^−7^	0.99 [0.76–1.29]	0.94	1.03·10^−5^	0.93 [0.71–1.21]	0.58	2.34·10^−4^	0.92 [0.71–1.20]	0.56	6.87·10^−4^
Q3	1.35 [1.04–1.77]	0.03		1.34 [1.03–1.75]	0.03		0.94 [0.73–1.22]	0.66		0.94 [0.73–1.22]	0.65	
Q4	1.13 [0.86–1.48]	0.37		1.11 [0.85–1.46]	0.44		1.06 [0.82–1.36]	0.65		1.05 [0.82–1.36]	0.69	
Q5	1.74 [1.34–2.24]	2.43·10^−5^		1.69 [1.29–2.22]	1.34·10^−4^		1.41 [1.10–1.82]	7.70·10^−3^		1.38 [1.07–1.80]	0.01	

Base model adjusted for sex, age at diagnosis and BMI and stratified by fasting glucose or HbA1c tertiles. Adjusted model includes sex, BMI, HDL, age at diagnosis, triglycerides, HbA1c at baseline, oral glucose lowering drugs, insulin use (for complication models) and eGFR (kidney function-related models) stratified by tertiles of glucose or HbA1c

### A high FG-CV is a risk factor for retinopathy, macrovascular complications and mortality

FG-CV and HbA1c-CV of the first interval (year 1–5 after diagnosis, N = 3963) were investigated as risk factors for diabetes-related complications during follow-up (median 8–9 years). A high FG-CV in the first interval was associated with proliferative retinopathy independent of mean fasting glucose (Table 2). The two highest quintiles were significant (HR_Q4_ = 2.35[1.22–4.51], *P *= 0.01, HR_Q5_ = 2.59[1.34–5.01], *P *= 4.56·10^−3^, Table 2), with the overall trend also significant (*P*_trend_ = 1.08·10^−3^). HbA1c-CV in the first interval was not associated with retinopathy (*P*_trend_ = 0.80).

In relation to kidney-related complications, FG-CV was a strong risk factor for microalbuminuria (HR_Q5_ = 1.63 [1.36–1.96], *P *= 1.33·10^−7^, *P*_trend_ = 4.23·10^−7^) and macroalbuminuria (HR_Q5_ = 2.89 [1.82–4.60], *P *= 7.40·10^−6^, *P*_trend_ = 2.98·10^−7^). The hazards for HbA1c-CV were generally lower and were only significant for microalbuminuria (HR_Q5_ = 1.38 [1.14–1.66], *P *= 7.11·10^−4^, *P*_trend_ = 1.43 ·10^−4^). eGFR stage did not show any consistent results for FG-CV or HbA1c-CV. The hazards for CKD stage did increase for FG-CV, but not HbA1c-CV, but were not significant (*P *> 0.05).

Higher FG-CV was associated (*P*_trend_ = 3.96·10^−3^) with a higher risk to develop macrovascular complications, with hazard ratios of 1.39 (95% CI 1.01–1.91, *P *= 0.04) for the third quintile, 1.43 (95% CI 1.02–1.94, *P *= 0.04) for the fourth quintile and 1.62 (95% CI 1.16–2.26, *P *= 4.28·10^−3^) for the highest quintile (Table 2). For HbA1c-CV, the effects were largely in the same direction, but only significant in the highest quintile (HR_Q5_ = 1.39 [1.01–1.91], *P *= 0.05, *P*_trend_ = 0.04).

Finally, a higher FG-CV was associated with a higher mortality risk for the three highest quintiles, but only the second (HR_Q2_ = 1.34[1.03–1.75], *P *= 0.03) and the highest quintile (HR_Q5_ = 1.69[1.29–2.22], *P *= 1.34·10^−4^) showed a significantly higher mortality risk (*P*_trend_ = 1.03·10^−5^, Table 2). For HbA1c-CV, the highest quintile was significantly associated with an increased risk of mortality (HR_Q5_ = 1.38[1.07–1.80], *P *= 0.01, *P*_trend_ = 6.87·10^−4^).

## Discussion

We investigated the differences in characteristics of people with low and high glycemic variability in people with type 2 diabetes. FG-CV across intervals increased with time, while HbA1c-CV remained largely stable and the correlation between the two decreased. Both a higher FG-CV and HbA1c-CV were characterized by an earlier disease onset, a higher BMI, lower HDL-C and higher triglycerides. Insulin use, and initiation was consistently higher in those with a high CV. Individuals with a high FG-CV in the first 5 years—but to a lesser extent for HbA1c-CV—showed an increased risk to develop retinopathy, macrovascular complications and were at increased risk of mortality, independent of mean FG or HbA1c, time since diagnosis and number of follow-up measurements.

A high HbA1c-CV and FG-CV was associated with an earlier disease onset, suggesting that those in which the disease onset is early in life suffer from a poorer glycemic control compared to those with a late onset [29].

Individuals with a high FG-CV in the first 5-years of their disease were at high risk to initiate insulin compared to those with a low FG-CV. A previous study showed that individuals with higher HbA1c variability were those on a more extensive treatment regimen, compared to those with a low HbA1c variability [20]. People with a high glycemic variability have been shown to be more often insulin deficient, which could be an explanation for their higher risk on insulin initiation [30].

### FG-CV over the first 5 years is a risk factor for micro- and macrovascular complications

FG-CV and to a lesser extent HbA1c-CV were risk factors for micro- and macrovascular complications. The number and timing of visits was the same in all study subjects these observations were. This in contrast to many previous studies that have looked at FG-CV and HbA1c as risk factors for diabetes-related complications. For microvascular complications, an increased risk was observed for retinopathy with increasing FG-CV. In two cross-sectional studies, contradicting results were observed between FG-CV and retinopathy outcome, but this may be due to the heterogeneity of the studies in terms of number of follow-up measurements and time since diagnosis [3, 31]. A meta-analysis on HbA1c variability did not find a relation with retinopathy, although this was based on only two studies and not based on variability of fasting glucose [32].

A high FG-CV in the first interval was associated with an increased risk of incident macrovascular complications, much stronger than HbA1c-CV. An increased risk for macrovascular complications has been observed in previous studies [6, 17], for example, FG-CV has been shown to be a risk factor for ischemic stroke in Taiwanese [33], but also with stroke and myocardial infarction in a German population [8]. Although we did see a relation in the fully adjusted models for incident CVD, a cross-sectional study did not find a relation for HbA1c-CV with CVD [34].

In terms of kidney-related complications, FG-CV and to a lesser extent HbA1c-CV were risk factors for micro- and macroalbuminuria. For eGFR-stages no consistent associations were found for both FG-CV and HbA1c-CV. In the CKD-stages a modest effect was seen, but this is likely driven by the stronger effects of the albuminuria. In a previous study, FG-SD—but not Hba1c-SD—was associated with microalbumuria [16]. Other studies have shown that the glycemic variability measures HbA1c-CV and SD were risk factors for chronic kidney disease with modest effect sizes [4, 35, 36]. In a meta-analysis of these studies HbA1c-SD was also identified as a risk factor for CKD [37]. Explanations for lack of association between CV and kidney-related complications are the complexity of the outcome, including differences in genetic background and medication use. In addition, earlier studies may have been confounded by diabetes duration or follow-up time. Since age is a very important predictor of kidney function decline, accounting for such age-related factors may also explain the lack of association in our study.

Finally, we observed FG-CV and to a lesser extend HbA1c-CV to be a risk factor for mortality. In previous studies similar mortality risks were observed [6, 9, 16, 38]. HbA1c-CV was only associated with mortality in the adjusted model, but not in the crude model.

The different distribution of individuals over quintiles in FG-CV vs HbA1c-CV and the subsequently different results for both measures, suggest that HbA1C-CV and FG-CV are not mutually exclusive. Fasting glucose levels reflect an individual’s ability to regulate glucose levels in absence of dietary glucose intake and is therefore independent of the dietary glucose intake itself. In previous studies it has been shown that postprandial glucose levels correlate better to HbA1c. Moreover, our results suggest that levels of variability of fasting glucose are of added value to solely measuring HbA1c. After all, a high FG-CV in the first 5 years after diagnosis was already indicative of a higher risk to develop retinopathy, macrovascular complications and mortality. Future studies should explore whether measures of glycemic variability over time can improve prediction of vascular complications in people with type 2 diabetes.

The strengths of our study include its large sample size with long follow-up duration, a deeply phenotyped cohort with highly standardized annual measurements, which allow us to compare the FG-CV with various outcomes across > 20 years of disease duration and follow-up. Another strength of our study is that the time between follow-up measurements is relatively constant with individuals visiting DCS once a year rather than most other studies where the frequency of sampling over years may vary considerably. A final strength of our study is that we aligned the visits based on time since diagnosis and the number of visits/measurements per interval.

A limitation of our study is the number of individuals varied across intervals. As such the individuals with a longer follow-up were generally those with an earlier disease onset. A second limitation is that intervals were aligned based on the date of diagnosis, while ideally one would use the date of disease onset. A final limitation is that the data is limited to visit-to-visit, while ideally one would also investigate the relation to continuous glucose monitoring.

FG-CV increased with time, while HbA1c-CV did not. This suggests that FG-CV adds information to HbA1c levels alone, particularly in people with a longer diabetes duration. A high FG-CV was associated with poorer health profile compared to those with low FG-CV, characterized by an earlier age of disease onset, a higher BMI, low HDL-C and higher triglycerides levels and individuals were more often current smokers. Individuals with a high FG-CV had a high risk for insulin initiation across the entire follow-up most likely related to more intensive treatment. Finally, FG-CV was a risk factor for retinopathy, macrovascular compilations and mortality independent of time since diagnosis, follow-up duration and mean glucose concentrations.

## Conclusion

Our results show that fasting glucose variability in the first 5 years—independent of mean glycaemia—is informative of current health status but may also be predictive of faster progression towards insulin and higher risk on complications. For HbA1c, the associations were weaker and less consistent. Our results suggest that FG-CV could serve as an important indicator for diabetes progression, in addition to classical measures such as HbA1c.

## Supplementary information

**Additional file 1: Figure S1.** Study setup. a) Flowchart of the individuals excluded from the analyses. b) Schematic representation of the calculation of variability in 5-year intervals.

**Additional file 2: Table S1.** Number of individuals per interval.

**Additional file 3: Table S2.** Characteristics of individuals across HbA1c-CV quintiles in the first interval.

**Additional file 4: Figure S2.** Comparison of FG-CV and HbA1c-CV across intervals. a) Median FG-CV across intervals. Line represents smoothed medians. Light blue, fasting glucose, dark blue HbA1c. b) Spearman correlation between FG-CV and HbA1c-CV across intervals. Line represents a linear regression line. c) Percentage of intervals compared to total number of individuals that ended up in a certain quintile, i.e. 100% means all intervals of an individual were assigned to that quintile. d) Overlap between FG-CV quintiles and HbA1c-CV quintiles.

**Additional file 5: Figure S3.** Characterization of individuals based on their HbA1c-CV. Unadjusted outcome against the five quintiles with the fifth quintile being the group with the highest FG-CV against (a) sex (% males) (b) age at diagnosis (c) BMI (d) % smokers (e) total cholesterol levels (f) HDL-C levels (g) triglycerides levels (h) DBP (i) SBP (j) eGFR. Lines represent loess smoothed levels over 5-year average values of outcomes.

**Additional file 6: Table S3.** Unadjusted and adjusted effect sizes of models with FG-CV for each quintile.

**Additional file 7: Table S4.** Unadjusted and adjusted effect sizes of models with HbA1c-CV for each quintile.

**Additional file 8: Table S5.** Odds ratios of medication use (metformin, SU, insulin) across intervals. The lowest quintile is used as the reference group.

**Additional file 9: Figure S4.** Glucose-lowering treatments across HbA1c-CV quintiles. a–e Percentage of individuals per quintile untreated (a), on metformin only (b), combination of metformin and SU (c), SU monotherapy (d) and insulin (e). f–h Odds ratios of the four highest quintiles versus the lowest quintile across time for (f) metformin (g) sulphonylureas and (h) insulin. Abbreviations: SU, sulphonylureas.

## Data Availability

Not applicable.

## Abbreviations

CKD: chronic kidney disease
CV: coefficient of variation
DBP: diastolic blood pressure
eGFR: estimated Glomerular Filtration Rate
FG: fasting glucose
GV: glycemic variability
HR: hazard ratio
Q: quintile
SBP: systolic blood pressure
SU: sulphonylurea derivatives
