# Supplementary figures and images for: Visit-to-visit variability of glycemia and vascular complications: the Hoorn Diabetes Care System cohort
Source: Cardiovasc Diabetol. 2019 Dec 12;18:170. doi: 10.1186/s12933-019-0975-1 (PMC6909524; doi:10.1186/s12933-019-0975-1)

a

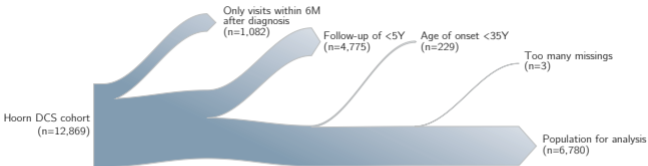

b

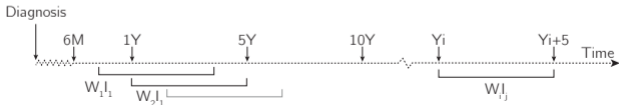

Supplement: Supplementary file 1 — Additional file 1: Figure S1. Study setup. a) Flowchart of the individuals excluded from the analyses. b) Schematic representation of the calculation of variability in 5-year intervals. [file 12933_2019_975_MOESM1_ESM.pdf]

a

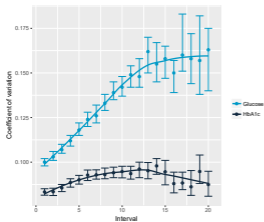

c

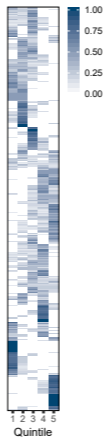

d

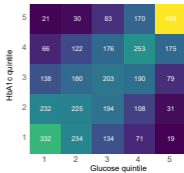

b

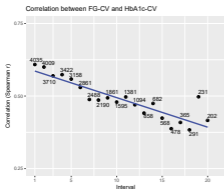

Supplement: Supplementary file 4 — Additional file 4: Figure S2. Comparison of FG-CV and HbA1c-CV across intervals. a) Median FG-CV across intervals. Line represents smoothed medians. Light blue, fasting glucose, dark blue HbA1c. b) Spearman correlation between FG-CV and HbA1c-CV across intervals. Line represents a linear regression line. c) Percentage of intervals compared to total number of individuals that ended up in a certain quintile, i.e. 100% means all intervals of an individual were assigned to that quintile. d) Overlap between FG-CV quintiles and HbA1c-CV quintiles. [file 12933_2019_975_MOESM4_ESM.pdf]

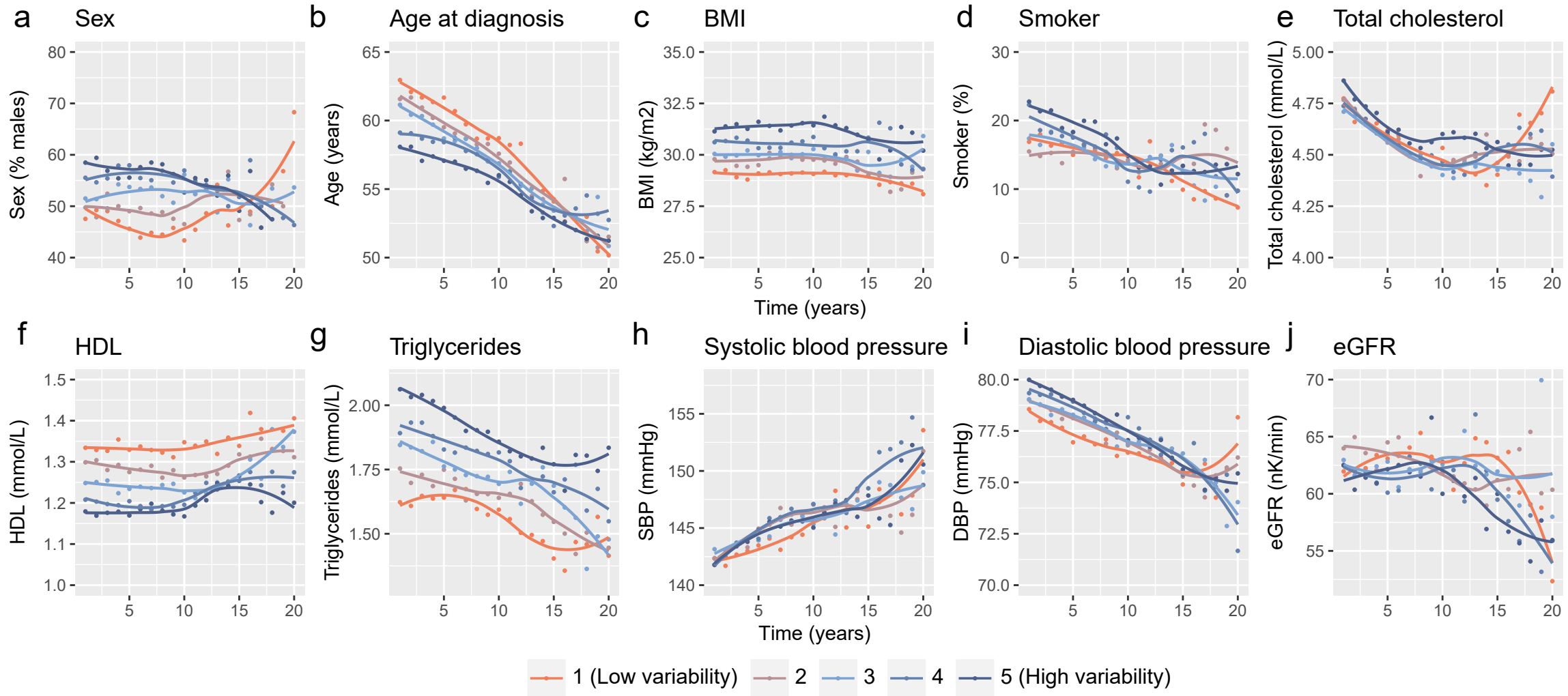

Supplement: Supplementary file 7 — Additional file 7: Table S4. Unadjusted and adjusted effect sizes of models with HbA1c-CV for each quintile. [file 12933_2019_975_MOESM7_ESM.pdf]

a

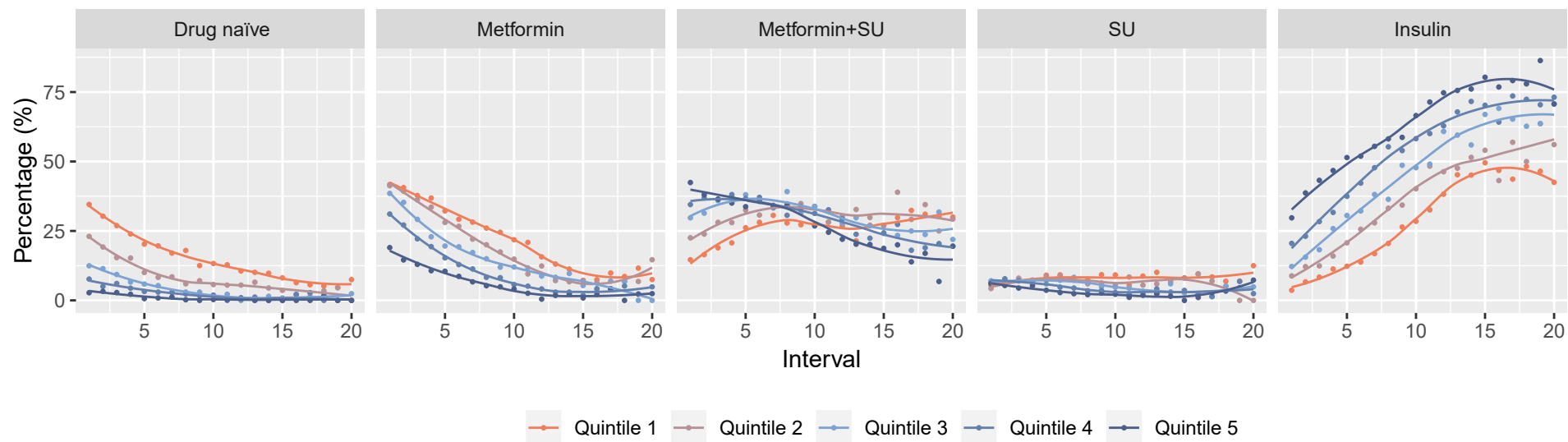

b

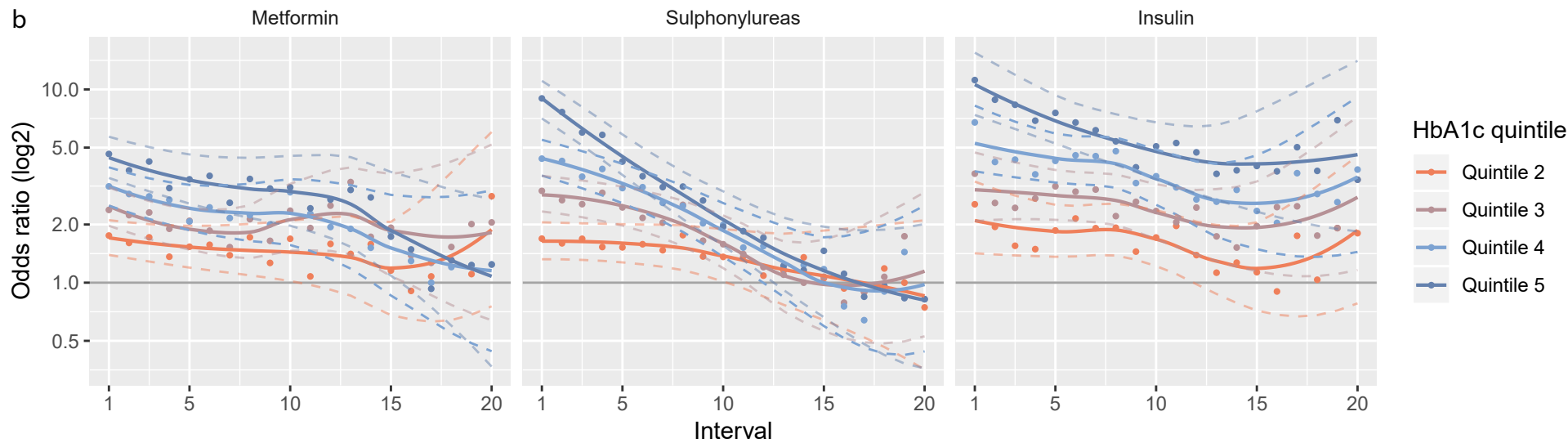

Supplement: Supplementary file 9 — Additional file 9: Figure S4. Glucose-lowering treatments across HbA1c-CV quintiles. a–e Percentage of individuals per quintile untreated (a), on metformin only (b), combination of metformin and SU (c), SU monotherapy (d) and insulin (e). f–h Odds ratios of the four highest quintiles versus the lowest quintile across time for (f) metformin (g) sulphonylureas and (h) insulin. Abbreviations: SU, sulphonylureas. [file 12933_2019_975_MOESM9_ESM.pdf]
