# Supplementary material for: Visit-to-visit variability of glycemia and vascular complications: the Hoorn Diabetes Care System cohort
Source: Cardiovasc Diabetol. 2019 Dec 12;18:170. doi: 10.1186/s12933-019-0975-1 (PMC6909524; doi:10.1186/s12933-019-0975-1)
Supplement: Supplementary file 2 — Additional file 2: Table S1. Number of individuals per interval. [file 12933_2019_975_MOESM2_ESM.docx]

| **Table S1. Number of individuals per interval** | |
| --- | --- |
| *Interval* | *#Individuals* |
| 1 | 3963 |
| 2 | 3983 |
| 3 | 3703 |
| 4 | 3396 |
| 5 | 3137 |
| 6 | 2822 |
| 7 | 2469 |
| 8 | 2168 |
| 9 | 1838 |
| 10 | 1588 |
| 11 | 1360 |
| 12 | 1080 |
| 13 | 847 |
| 14 | 671 |
| 15 | 556 |
| 16 | 468 |
| 17 | 368 |
| 18 | 293 |
| 19 | 222 |
| 20 | 203 |
